# Impaired memory is more closely associated with brain beta-amyloid than leukoaraiosis in hypertensive patients with cognitive symptoms

**DOI:** 10.1371/journal.pone.0191345

**Published:** 2018-01-30

**Authors:** Eric E. Smith, Alona Muzikansky, Cheryl R. McCreary, Saima Batool, Anand Viswanathan, Bradford C. Dickerson, Keith Johnson, Steven M. Greenberg, Deborah Blacker

**Affiliations:** 1 Department of Clinical Neurosciences, University of Calgary, Calgary, Canada; 2 Hotchkiss Brain Institute, University of Calgary, Calgary, Alberta, Canada; 3 Department of Biostatistics, Massachusetts General Hospital, Boston, Massachusetts, United States of America; 4 Department of Radiology, University of Calgary, Calgary, Alberta, Canada; 5 Department of Neurology, Massachusetts General Hospital, Harvard Medical School, Boston, Massachusetts, United States of America; 6 Department of Psychiatry, Massachusetts General Hospital, Harvard Medical School, Boston, Massachusetts, United States of America; 7 Department of Epidemiology, Harvard T.H. Chan School of Public Health, Boston, Massachusetts, United States of America; Monash University, AUSTRALIA

## Abstract

**Background:**

Hypertension is the strongest modifiable risk factor for subcortical ischemic changes and is also a risk factor for Alzheimer’s dementia. We used neuroimaging to investigate the pathological basis of early cognitive symptoms in patients with hypertension.

**Methods:**

In this cross-sectional cohort study 67 patients age >60 years with hypertension and Clinical Dementia Rating scale score of 0.5 without dementia, and without history of symptomatic stroke, underwent MRI for measurement of subcortical vascular changes and positron emission tomography (PET) scan with Pittsburgh Compound B (PiB-PET) to detect beta-amyloid deposition. These imaging measures were related to neuropsychological tests of memory, executive function and processing speed.

**Results:**

Mean age was 75.0 (standard deviation, SD, 7.3). Mean neuropsychological Z scores were: episodic memory -0.63 (SD 1.23), executive function -0.40 (SD 1.10), processing speed -0.24 (SD 0.88); 22 of the 67 subjects met criteria for mild cognitive impairment (MCI) and the remaining 45 subjects had subjective cognitive concerns only. In multivariable models adjusting for age and years of education, each 0.1 unit increase in mean cortical PiB-PET binding was associated with 0.14 lower mean Z score for episodic memory (95% CI -0.28 to -0.01). This means that for every 0.1 unit increase in mean cortical PiB-PET, episodic memory was 0.14 standard deviations lower. White matter hyperintensity volume, silent brain infarcts and microbleeds were not associated with neuropsychological test scores.

**Conclusions:**

Episodic memory was prominently affected in hypertensive participants with MCI or subjective cognitive concerns, and was associated with PiB-PET binding. This suggests a prominent role for Alzheimer pathology in cognitive impairment even in hypertensive participants at elevated risk for vascular cognitive impairment.

## Introduction

Hypertension is a risk factor for vascular dementia as well as dementia due to clinically probable Alzheimer’s disease (AD).[1–3] Hypertension is also a risk factor for mild cognitive impairment (MCI),[4] and may also increase the risk of conversion from MCI to dementia.[5] More recently, research has focused on patients with subjective cognitive concerns (SCC) but with normal range performance on cognitive testing.[6] However, few studies have used neuroimaging biomarkers to investigate the underlying neuropathological basis of MCI or SCC in patients with hypertension. Because hypertension is the strongest and most common risk factor for subcortical ischemic pathology,[7] it is likely that hypertensive patients with MCI or AD will harbor relatively more vascular and mixed pathology compared to other MCI cases.

To study the pathogenic basis of cognitive symptoms in the setting of hypertension, we recruited subjects with hypertension and Clinical Dementia Rating (CDR)[8] of 0.5 without dementia and used neuroimaging markers to infer the presence of AD pathology (by Pittsburgh Compound B positron emission tomography [PiB-PET]) or vascular pathology (by measuring the volume of white matter hyperintensities [WMH] of presumed vascular origin). In contrast to studies focusing on prodromal AD we did not exclude participants with higher burden of subcortical ischemic disease (silent brain infarcts and WMH) in order to recruit a study population more representative of community patients with hypertension. We hypothesized that PiB-PET binding would correlate with poor performance on episodic memory tasks while higher WMH volume would correlate with poor performance on tasks of executive function and psychomotor processing speed.

## Methods

### Study population

Study participants were recruited from 2009 to 2013 at a National Institute on Aging Alzheimer’s Disease Research Center (ADRC), from among participants being followed longitudinally across the spectrum of cognitive impairment. These individuals were initially recruited from an affiliated memory disorders unit, from local medical clinics, and from the community. Inclusion criteria were: age >60, history of hypertension (defined as reported diagnosis of hypertension and use of one or more antihypertensive medications), and CDR rating of 0.5 indicating substantiated cognitive concerns. Exclusion criteria were a diagnosis of dementia (defined using DSM-IV criteria), history of symptomatic stroke (although silent brain infarction was not an exclusion), history of other central nervous system diseases, serious medical or psychiatric illness that would interfere with study participations, or contraindications to MRI. A total of 67 participants were recruited and completed all baseline study procedures.

### Study measurements

At the baseline study visit, data were collected on age, demographics, medical history, and antihypertensive medication use. The CDR was determined based on a semi-structured interview conducted by a behavioral neurologist or geriatric psychiatrist.[8, 9] Blood pressure was measured once in the right arm while sitting, and once after standing for 5 minutes.

We selected the neuropsychological tests from those included in the National Institute on Aging (NIA) Alzheimer’s Disease Centers’ Uniform Data Set (ADC UDS)[10] as well as additional tests that, based on earlier work, were sensitive to change in episodic memory, executive function and processing speed.[11] To express individual test results as z scores, we derived our own local normative data using 303 cognitively normal (CDR 0) participants in the local ADRC Longitudinal Cohort. Prior to any data analysis, we grouped test scores into three cognitive domains—episodic memory, executive function and psychomotor processing speed—based on the known cognitive properties of each test. “Episodic memory” was calculated as the average of the Wecshler Logical Memory II and California Verbal Learning Test-II Long Delay Free Recall z scores; “executive function” was the average of fluency (consisting of equally weighted semantic fluency [animal and vegetable naming] and letter fluency [“F”, “A”, and “S” words] Z scores) and the z score of the logarithmic transformation of Trail Making B time minus Trail Making A time; and “processing speed” was the average of Trail Making A and Digit Symbol Substitution test z scores.

The z scores of our 67 CDR 0.5 participants where then expressed in relation to the normative data collected at our site. A psychiatrist (DB), blinded to PiB-PET and WMH volume results, reviewed the clinical history, CDR and neuropsychological test results to classify participants as having either MCI or SCC. All participants had subjective memory concerns as quantified by a structured interview to determine the CDR. MCI was defined using National Institute on Aging-Alzheimer’s Association criteria.[12] A cognitive domain score lower than -1.0, indicating performance more than one standard deviation below normal, was used as the criterion for objective evidence of cognitive impairment to support a diagnosis of MCI. Participants with subjective memory concerns (CDR 0.5) without MCI were defined as having SCC.

MRI was performed on a single Siemens 3.0 T Trim Trio (Siemens Healthcare GmbH; Erlangen, Germany). Sequences included: 3D T1-weighted magnetization prepared rapid gradient echo (MP-RAGE) (TR/TE = 2300/2.98 ms, inversion time = 900 ms, flip angle = 9° and 1 mm^3^ isotropic resolution), 3D fluid-attenuated inversion recovery (FLAIR) (TR/TE = 6000/455 ms, inversion time = 2100 ms, flip angle = 120° and 1 mm^3^ isotropic resolution), dual-echo T2-weighted and proton density-weighted (TR/TE1/TE2 = 3000/11/99 ms, echo train length = 7, 0.9375 × 9.9375 × 3.0 mm^3^ voxels) and susceptibility-weighted imaging (SWI) (TR/TE 27/20 ms, flip angle = 15°, 0.8984 x 0.8984 x 1.5 mm^3^ voxels). WMH, silent brain infarcts and microbleeds were defined and measured according to consensus Standards for Reporting Vascular Changes on Neuroimaging (STRIVE).[13] A radiologist identified silent brain infarcts and microbleeds. WMH volume was measured by a single rater using custom-designed Quantomo software (Cybertrials, Inc; Calgary, Canada). In brief, the rater (SB) placed seeds in regions of WMH, then lesion boundaries were automatically detected based on a three-dimensional threshold-based region growing segmentation method, based on noise-filtered and bias field corrected FLAIR data. All WMH masks were reviewed by the rater for accuracy; manual editing tools allowed correction of any mislabeling.[14] In a separate analysis of 30 scans from patients with TIA or mild ischemic stroke analyzed by three raters, the inter-rater intraclass correlation coefficient (ICC) was 0.97 and the intra-rater ICC was 0.99. This study’s rater (SB) was certified to analyze WMH based on an ICC of 0.99 (95% confidence interlal 0.97–0.99). To account for differences in participant head size, WMH was analyzed as the percent of intracranial volume (ICV) occupied by WMH. ICV was determined by processing the data using Freesurfer version 5.0.[15] MRI measurements were made blinded to clinical data and PiB-PET results. We have previously shown good reliability of these measurements.[14, 16]

PiB PET data were acquired using a Siemens HR+ (Siemens CTI, Knoxville, TN), and each frame was evaluated to verify adequate count statistics and absence of head motion. The Logan graphical analysis method[17] with cerebellar cortex as the reference tissue input function was used to evaluate specific PiB retention expressed as the distribution volume ratio (DVR) as previously described.[18] Mean cortical PiB retention (PiB) was calculated in an aggregate over all cortical ROIs.add17 PiB retention was analyzed as both a continuous and categorical variable; a PiB DVR cut-point was used to classify individuals into either PiB+ or PiB- groups, as in previous studies.[19]

### Statistical analysis

Neuropsychological test scores and cognitive domain scores were approximately normally distributed and summarized as means and standard deviations, with the exception that Trail Making B time and Trail Making B time minus Trail Making A time were right-skewed, and therefore logarithmically transformed to a normal distribution before conversion to z scores. PiB-PET DVR and WMH are summarized as medians and interquartile ranges (IQRs). For comparability with previous studies, mean values are also tabulated. The Pearson correlation coefficient was used to test correlations between neuroimaging markers and cognitive domain scores. Because WMH was very right-skewed, it was log-transformed to a more normal distribution before testing correlations.

**Fig 1:**
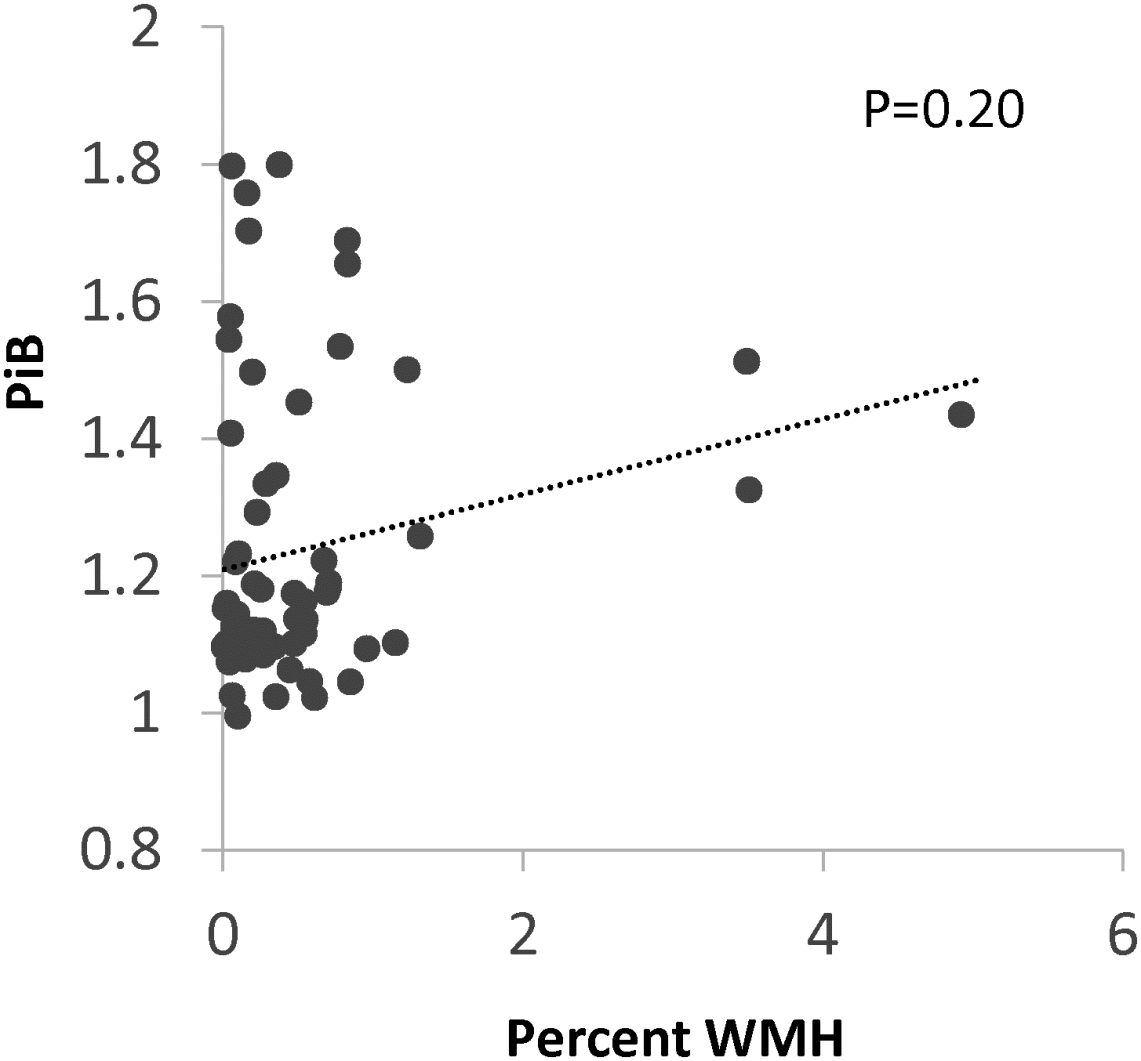
Association between PiB and WMH. PiB; mean cortical Pittsburgh compound B binding, expressed as the distribution volume ratio; WMH, MRI white matter T2 hyperintensity. WMH is analyzed as the percent of intracranial volume, log-transformed to a more normal distribution. The best fit line and p value are from unadjusted linear regression.

To determine whether each neuroimaging marker (PiB-PET and WMH) was associated with cognitive domain scores, we first evaluated separate linear regression models for each marker and domain score. Cognitive domain scores were used as the dependent variables, while neuroimaging markers were entered as independent variables. Based on prior literature, all models included age and education as covariates. Sex was not associated with cognitive performance in this study and therefore it was not included in the models. PiB was analyzed as continuous variable for the primary analysis, but in a secondary analysis was analyzed as PiB positive (defined as global DVR ≥1.25) vs. PiB negative instead. Next, we repeated the models including both PiB and WMH in the same model. Finally, we tested interactions between PiB (dichotomized as positive vs. negative) and WMH in models. To generate the plots in Fig 1, the age- and education-independent relationships between cognitive domain scores and neuroimaging markers, we first modeled cognitive domain scores as a function of age and education. Then, we modeled neuroimaging markers as a function of these residuals, adjusting for age and education in each model. We defined confounding based on a change-in-estimate criterion of ≥10% change in the model covariate of interest.[20] Statistical testing was done using SAS version 9.3 (Cary, NC). A *p* value of ≤0.05 was considered significant; we did not adjust the *p* value threshold for multiple comparisons because the study hypotheses were exploratory, but pre-specified.

#### Protocol approval and patient consent

The study was approved by institutional review boards of Massachusetts General Hospital and the University of Calgary. Participants provided written informed consent.

## Results

Characteristics of the study cohort are shown in Table 1. Mean age was 75.0 years, 49% were women, and the mean number of years of education was 16.1. Mean systolic blood pressure was 134±14 mmHg and mean diastolic blood pressure was 72±10 mmHg. Despite having a history of treated hypertension, high measured blood pressures (SBP ≥140 mmHg or DBP ≥90 mmHg) were present in 28/67 (42%).

**Table 1:** Characteristics of the study population.

Characteristic	Overall Cohort N = 67	SCC N = 45	MCI N = 22	*P* value
Age	75.0 ± 7.3	75.0 ± 6.8	75.0 ± 8.3	0.99
Female	33 (49%)	22 (49%)	11 (50%)	0.99
Education (years)	16.1 ± 2.6	16.5 ± 2.6	15.3 ± 2.4	0.06
Congestive heart failure	1 (1%)	0 (0%)	1 (5%)	0.33
Coronary artery disease	0 (0%)	0 (0%)	0 (0%)	0.99
Atrial fibrillation	8 (12%)	6 (13%)	2 (9%)	0.99
Diabetes	12 (18%)	8 (18%)	4 (18%)	0.99
Hypercholesterolemia	46 (69%)	33 (73%)	13 (59%)	0.27
Current smoker	6 (8%)	5 (11%)	1 (5%)	0.66
Mean systolic BP	134 ± 14	134 ± 13	135 ± 16	0.68
Mean diastolic BP	72 ± 10	72 ± 10	72 ± 10	0.91
Global PiB	1.14 [1.09–1.33]	1.14 [1.09–1.23]	1.16 [1.09–1.44]	0.53
PiB ≥1.25	20 (30%)	11 (24%)	9 (41%)	0.26
WMH, median (cm^3^)	3.4 [1.6–9.3]	4.2 [1.7–8.6]	2.0 [0.8–9.3]	0.11
WMH, mean (cm^3^)	7.7 ± 13.2	8.0 ± 14.9	7.3 ± 22.6	0.84
WMH as percent ICV, median	0.23% [0.10–0.58%]	0.27% [0.12–0.55%]	0.16% [0.05–0.58%]	0.11
WMH as percent ICV, mean	0.51 ± 0.83%	0.52 ± 0.73%	0.49 ± 1.03%	0.88
Silent brain infarcts	12 (18%)	8 (18%)	4 (18%)	0.99
Microbleeds	15 (22%)	13 (29%)	2 (9%)	0.99
Microbleed pattern				0.31
1 lobar CMB	10 (15%)	9 (20%)	1 (5%)	
>1 lobar CMB	2 (3%)	2 (4%)	0 (0%)	
Deep or mixed CMBs	3 (4%)	2 (4%)	1 (5%)	

BP, blood pressure; PiB, mean cortical PET Pittsburgh Compound B binding; WMH, white matter hyperintensity; CMB, cerebral microbleed. Values are percentages, mean ± standard deviation, or median [25^th^ percentile-75^th^ percentile]. There were no missing data.

Neuropsychological test score performance, including raw scores and z scores relative to local norms, are shown in the Supporting Information. Compared to cognitively normal individuals from the demographically similar ADRC Longitudinal Cohort sample, our participants had significantly lower scores. Mean episodic memory domain score was -0.63±1.23, executive function domain score was -0.40±1.10, and processing speed domain score was -0.37±0.88 (all p<0.01 for comparison with our normal controls). MCI was diagnosed in 22/67 (33%), the remainder were classified as subjective cognitive concerns.

Median global PiB-PET DVR was 1.14 (interquartile range, IQR, 1.09–1.33), and 20/67 (30%) had DVR above the 1.25 cut-off defined as “Pib-positive” for this study (Table 1). There were 12/67 (18%) with silent brain infarcts and 15/67 (22%) with microbleeds. There was no correlation between the PiB-PET DVR and WMH (r = 0.16, p = 0.20; Fig 1). Compared to participants with subjective cognitive concerns, participants with MCI did not differ in PiB-PET DVR (median 1.28, IQR 1.09–1.44 vs. 1.22, IQR 1.09–1.23, p = 0.53), percent PiB-PET positive (9/22, 41%, vs. 11/45, 24%, p = 0.25), or percent WMH volume (0.16%, IQR 0.05–0.58% vs. 0.27%, IQR 0.12–0.55%, p = 0.11). Among this study population with a known history of hypertension, there was no correlation between measured SBP or DBP and WMH, PiB-PET DVR or cognitive domain scores (p>0.20 for all comparisons).

In univariate analyses there was no relationship between PiB (analyzed as a continuous variable) and episodic memory (r = -0.16, p = 0.19), executive function (r = -0.18, p = 0.14) or speed (r = -0.15, p = 0.22); and no relationship between WMH and memory (r = 0.23, p = 0.06), executive function (r = 0.03, p = 0.81) or speed (r = -0.04, p = 0.63). However, higher PiB-PET DVR was associated with lower episodic memory score after adjusting for age and education (Table 2; p = 0.04). There was evidence that the association between PiB-PET DVR and memory was confounded by age and education, because the beta coefficient changed from -0.095 (95% CI -0.236 to 0.047) in univariate regression to -0.14 in multivariable adjusted regression (95% CI -0.28 to -0.01; Table 2). Additional exploratory analyses showed that the confounding was largely accounted for by age, with higher age being associated with higher memory in the fully adjusted model (each additional year of age was associated with 0.055 higher score on memory, 95% CI 0.016 to 0.095). There were no associations between higher WMH and any cognitive domains (Table 2).

**Table 2:** Multivariable models of cognition somain scores.

Exposure	Outcome	Estimated change in mean Z score	95% CI	*P* value
PiB-PET per 0.1 increase	Memory	**-0.14**	**-0.28 to -0.01**	**0.04**
	Exec Function	-0.12	-0.24 to 0.01	0.07
	Speed	-0.07	-0.17 to 0.04	0.21
WMH per increase of 1% of ICV	Memory	-0.02	-0.40 to 0.36	0.91
	Exec Function	-0.16	-0.50 to 0.18	0.35
	Speed	-0.04	-0.32 to 0.24	0.77

Results of six separate models for each cognitive domain (dependent variable) and neuroimaging marker (predictor variable). Models are also adjusted for age and years of education. The estimated change in mean Z score is equivalent to the number of standard deviations from the age- and education-adjusted mean. When including both PiB-PET and WMH in the same models the findings were the same, with the only positive finding being that PiB PiB-PET was associated with memory score independent of WMH volume (beta coefficient -0.14, 95% confidence interval -0.28 to -0.01).

**Fig 2:**
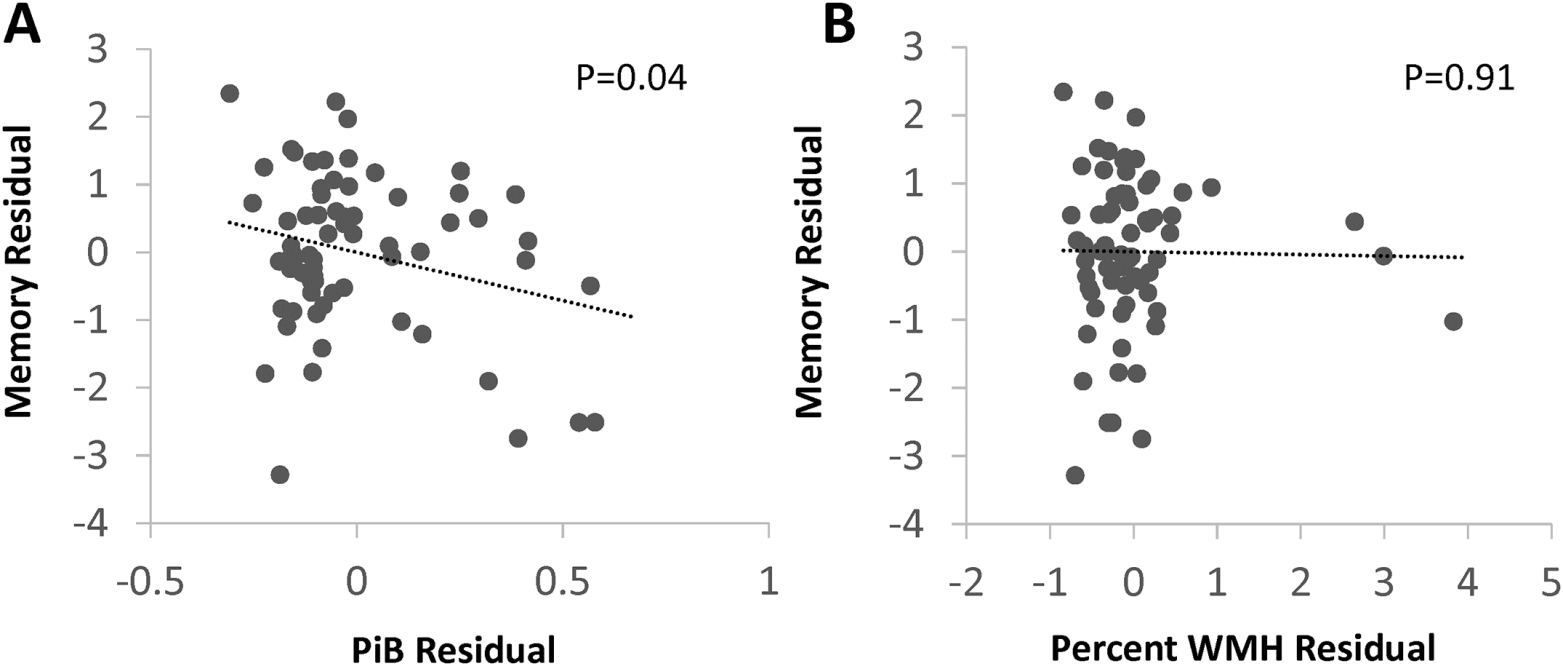
Associations of episodic memory score with PiB and WMH. PiB; mean cortical Pittsburgh compound B binding, expressed as the distribution volume ratio; WMH, MRI white matter T2 hyperintensity. Plots generated from models adjusted for age and education showed an independent relationship between lower memory and higher PiB (-0.14 lower memory Z score for each 0.1 increase in PiB distribution volume ratio, 95% confidence interval -0.28 to -0.01) but no relationship with WMH.

**Fig 3:**
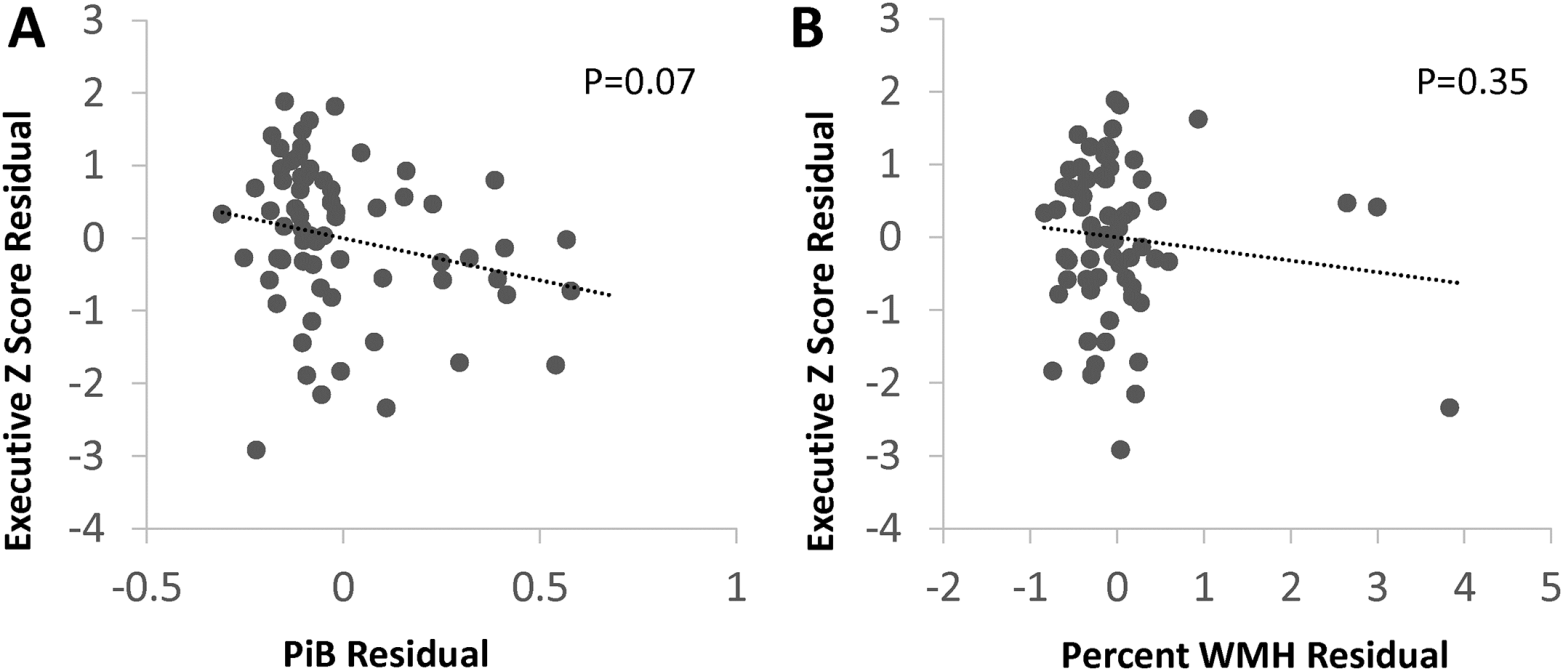
Associations of executive function score with PiB and WMH. PiB; mean cortical Pittsburgh compound B binding, expressed as the distribution volume ratio; WMH, MRI white matter T2 hyperintensity. Plots generated from models adjusted for age and education showed a non-significant trend toward lower executive function with higher PiB (-0.12 change per 0.1 unit increase in PiB distribution volume ratio, 95% CI -0.24 to 0.01) but no relationship with WMH.

**Fig 4:**
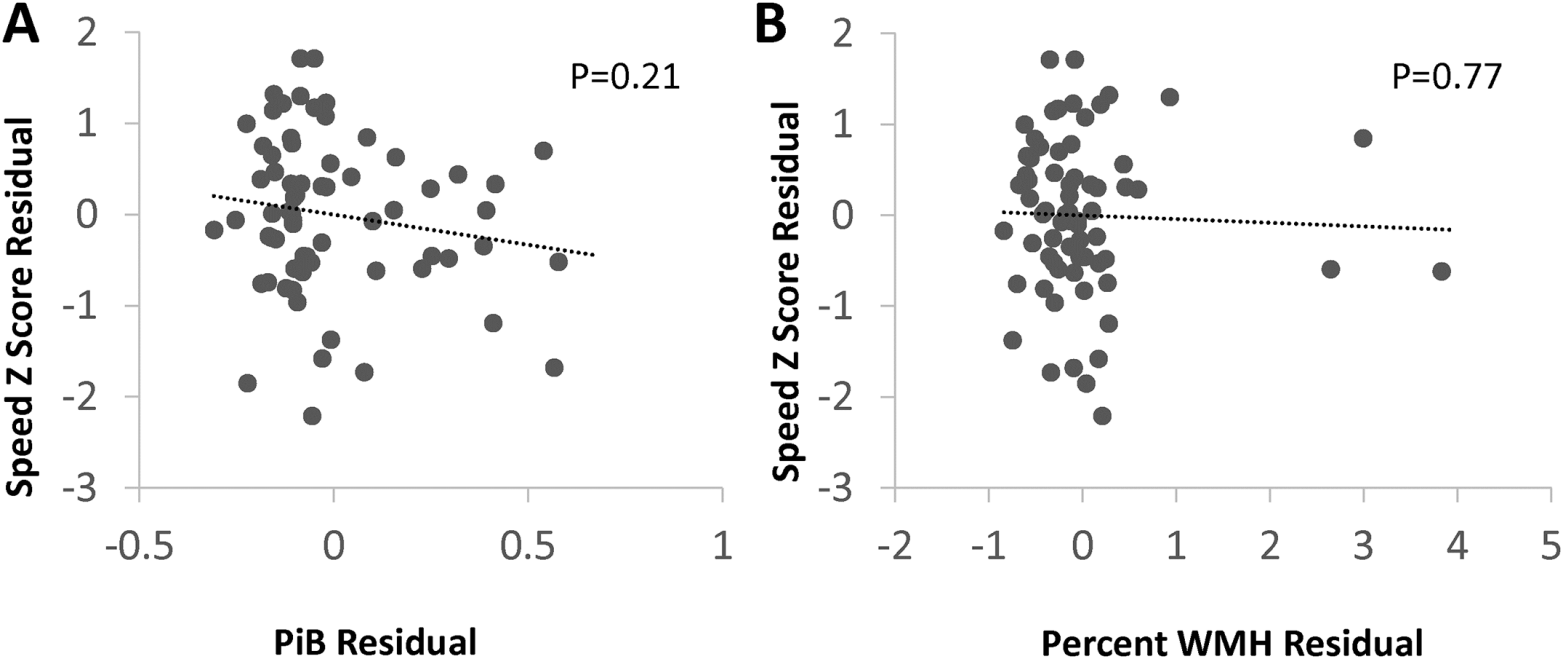
Associations of processing speed score with PiB and WMH. PiB; mean cortical Pittsburgh compound B binding, expressed as the distribution volume ratio; WMH, MRI white matter T2 hyperintensity. Plots generated from models adjusted for age and education showed no associations between PiB or WMH and processing speed.

Scatterplots of the age- and education-independent relationships between PiB and WMH and cognitive domains are shown in Figs 2–4. In additional analyses, we modeled each cognitive domain score including both PiB-PET DVR and WMH as predictors, and found similar relationships between the neuroimaging markers and cognitive domain scores—PiB-PET DVR was associated with lower episodic memory, with no evidence of confounding by WMH (the beta coefficient for PiB-PET DVR, -0.14, was the same as the model without WMH). We did not find evidence of an interaction between PiB-PET DVR and WMH on any cognitive domain (interaction *p* values >0.20), such the relationship between PiB-PET DVR and cognition did not differ in patients with high WMH compared to those with low WMH.

Finally, we analyzed the effects of blood pressure control on neuroimaging markers and cognitive domains. Compared to patients with measured blood pressure <140/90 mmHg (n = 39; mean SBP 125±10 mmHg and mean DBP 69±9 mmHg), patients with elevated blood pressure (n = 28; mean SBP 147±8 mmHg and mean DBP 75±10 mmHg) were older (mean age 77.6±5.3 years vs. 73.2±8.0; p = 0.009) and had non-significantly higher median WMH as a percent of intracranial volume (median 0.39% [interquartile range 0.18%-0.68%] vs. 0.15% [interquartile range 0.10%-0.49%]; p = 0.07). However, there were no significant differences in PiB-PET DVR, the proportion that were PiB-PET positive (based on the cut-off of 1.25), or in any of the three cognitive domains (p>0.20 for all comparisons).

## Discussion

In this study of participants with hypertension and either MCI or subjective cognitive concerns, a marker of beta-amyloid retention, PiB-PET, was associated with worse episodic memory but a marker of subcortical ischemic injury, WMH, was not associated with performance in any cognitive domain. The association of episodic memory with higher PiB-PET became apparent only after accounting for the effects of age and education in our multivariable model. The association was of borderline statistical significance and would no longer be significant if the p value was adjusted for comparisons with three distinct cognitive outcomes. Therefore, our findings should be considered exploratory and warrant confirmation in other studies. However, our findings reinforce the need to consider Alzheimer pathology as a cause of memory concerns, even in patients with hypertension and WMH.

Mean cognitive performance in the study participants was lower than local normative controls in all domains, but not markedly so. This reflects that our study entry criteria (CDR 0.5 without dementia) was designed to capture a population of mildly impaired persons with either MCI or subjective concerns. We chose to include participants with only subjective cognitive concerns as well as those meeting criteria for MCI, so that the study sample would reflect the entire spectrum of cognitive concerns without dementia. Evolving literature suggests that higher degrees of subjective concerns are associated with greater likelihood of positive PiB-PET,[21] and that some patients with subjective concerns are at risk for future cognitive decline.[22] In our study population, the volume of WMH and frequency of silent brain infarcts and microbleeds were higher than expected from population-based studies,[23] reflecting the entry criteria for hypertension. Even so, there were few participants with very high WMH volumes which may be one reason why we failed to identify a distinct cognitive pattern associated with WMH.

It is important to recognize that our study findings do not suggest that WMH are benign and not associated with cognitive impairment. A large body of literature shows that WMH are associated with worse cognition in the general population and patients with stroke.[24, 25] By design, we did not include a cognitively normal control group with neuroimaging whose WMH volume could be compared with our study participants. Our results only show that WMH are not associated with a specific pattern of cognitive impairment in patients with hypertension across the spectrum of mild cognitive concern and impairment.

There are few studies that have used amyloid PET to distinguish the independent contributions of amyloid and vascular pathology to cognitive impairment. Concordant with our findings, a study of Korean patients with clinical vascular dementia due to severe subcortical ischemic changes showed that PiB-PET positivity was common (14/45 patients) and that PiB-PET positivity was associated with worse memory performance.[26] Similarly, another study of 67 patients with vascular MCI found that the 22 PiB-PET positive patients had worse impairments in multiple cognitive domains.[27] A study of 168 cognitively normal elderly with no cognitive concerns (CDR 0) found that higher PiB-PET amyloid was specifically associated with worse episodic memory, as in our study of participants with mild concerns or impairment, but that WMH was associated most prominently with executive function.[28]

Our study findings contrast with these others in that WMH was not associated with a specific cognitive profile. In contrast to the prior studies of vascular dementia and MCI,[26, 27] our study population had lower WMH burden. While the mean WMH in our study was higher than in similar aged participants in the population-based Framingham study (0.50% vs. 0.098% and 0.129% for 72–96 year old men and women, respectively)[22] it was much lower than in the study of subcortical ischemic vascular MCI (7.7 cm3 vs. 34.9 cm3)[24], probably because very extensive WMH was one of the criteria used to define subcortical ischemic disease. In contrast to the prior study of normal cognition,[28] participation in our study was conditional on having cognitive concerns and it is possible that unmeasured factors could have accounted for cognitive impairments in patients with lower WMH burden, which would tend to obscure the relationship between WMH and cognition. These differences in WMH burden and cognitive symptoms may explain the different results between studies.

The prevalence of PiB-PET positive patients in our study was relatively low, reflecting the lower prevalence of PiB retention in SCC patients (24%) compared to MCI patients (41%), consistent with other studies.[29] Additionally, the low prevalence of PiB retention could reflect a higher prevalence of cerebrovascular disease as a competing cause of cognitive concerns and impairment in this study population with hypertension.

Currently, there is controversy as to whether cerebrovascular disease contributes to the deposition of beta-amyloid. Although one study found greater PiB-PET increases in patients with higher WMH[30] and another found a relationship between higher WMH and higher PiB-PET retention in apolipoprotein E ε4 carriers only,[31] our study findings are similar to the largest study in cognitively normal elderly which found that WMH and PiB-PET were not correlated.[32] Except in the specific context of cerebral amyloid angiopathy,[33] it seems likely that beta-amyloid deposition and WMH accrue as part of independent processes.

Our findings should be interpreted in light of several limitations. The sample size was insufficient to detect modest differences in cognitive patterns between neuroimaging markers; future, larger studies are needed to determine whether WMH may be association with more modest differences. The study was cross-sectional; however, we are currently following the study participants to determine the associations between baseline neuroimaging markers and risk for longitudinal cognitive decline and conversion to dementia. By design, our study did not include physiological measurements of hypertension severity in other organs (*e*.*g*. the degree of left ventricular hypertrophy), which could also be correlated with brain injury and cognitive impairment. Most of the participants in our study had controlled hypertension and did not have clinical evidence of significant hypertensive end-organ damage outside the brain. We pre-specified WMH volume as our marker of subcortical vascular ischemic injury because it is common, can be measured quantitatively and is known to be associated with cognitive impairment. However, the pathophysiology of WMH is uncertain and may be heterogenous.[7] Finally, our sample is a convenience sample, including individuals recruited from a memory clinic, from other clinical settings, and from the community, so the generalizability of our findings to the general population is limited.

Our finding that more than one quarter of our participants, all over 60 with hypertension and cognitive concerns or impairment are PiB-PET positive highlights the need to consider AD pathology (and potentially a diagnosis of MCI due to Alzheimer’s disease or late preclinical AD) as a common cause of cognitive impairment and cognitive concerns, even in patients with hypertension or other risk factors for vascular cognitive impairment. Where appropriate, AD biomarker testing, such as with PET amyloid imaging or cerebrospinal fluid analysis, could be considered for such patients with persistent, unexplained MCI.[34] Although our hypertensive participants with worse episodic memory were more likely to have Alzheimer pathology, we failed to find a pattern of cognitive impairment that suggested the presence of subcortical ischemic pathology. Because cognitive evaluation alone is not sufficient to identify hypertensive patients with subcortical ischemic pathology, neuroimaging will be essential—as recommended by American Heart Association/American Stroke association criteria for diagnosis of vascular cognitive impairment[35]—to identify vascular brain lesions that would prompt additional diagnostic work-up (e.g. for cause of silent brain infarction) and management for secondary prevention of cerebrovascular disease.

## Supporting information

S1 Table Mean neuropsychological test scores for individual tests. *Because of right skewed deviations, raw scores were logarithmically transformed prior to calculating Z scores. SD, standard deviation.
(DOCX)
