# Supplementary material for: Impaired memory is more closely associated with brain beta-amyloid than leukoaraiosis in hypertensive patients with cognitive symptoms
Source: PLoS One. 2018 Jan 30;13(1):e0191345. doi: 10.1371/journal.pone.0191345 (PMC5790236; doi:10.1371/journal.pone.0191345)
Supplement: S1 Table — *Because of right skewed deviations, raw scores were logarithmically transformed prior to calculating Z scores. SD, standard deviation. (DOCX) [file pone.0191345.s001.docx]

**SUPPLEMENTARY RESULTS**

**S1 Table. Mean Neuropsychological Test Scores for Individual Tests**

|  |  | **Raw Scores** | | **Normalized Z Scores** | |
| --- | --- | --- | --- | --- | --- |
| **Variable** |  | **Mean** | **SD** | **Mean** | **SD** |
| Logical Memory II |  | 12.7 | 4.6 | -0.32 | 1.40 |
| CVLT Long Delay Free Recall |  | 8.8 | 4.2 | -0.94 | 1.47 |
| No. of Animals |  | 19.2 | 5.1 | -0.34 | 1.01 |
| No. of Vegetables |  | 13.5 | 3.9 | -0.55 | 0.96 |
| FAS |  | 45.4 | 13.8 | -0.30 | 1.04 |
| Trails A time |  | 31.7 | 10.9 | -0.08 | 1.00 |
| Trails B time |  | 93.7 | 57.4 | -0.41* | 1.45 |
| Trails B minus A |  | 62.0 | 51.6 | -0.41* | 1.36 |
| Digit Symbol |  | 45.2 | 9.9 | -0.39 | 0.97 |

Table legend: *Because of right skewed deviations, raw scores were logarithmically transformed prior to calculating Z scores. SD, standard deviation.
